# Host–Pathogen Interaction 3.0

**DOI:** 10.3390/ijms232112811

**Published:** 2022-10-24

**Authors:** Andreas Burkovski

**Affiliations:** Microbiology Division, Friedrich-Alexander-Universität Erlangen-Nürnberg, 91054 Erlangen, Germany; andreas.burkovski@fau.de; Tel.: +49-9131-85-28086

Microorganisms can interact with plants, animals and humans in many different ways, e.g., beneficially as symbionts, indifferently as commensals or harmfully as pathogens. Today, a wide variety of molecular and cell biology tools, including advanced microscopy and -omics techniques, allow us to study these interactions at a molecular level. It is the dedicated aim of this serial of Special Issues, i.e., “Host–Pathogen Interaction”, Host–Pathogen Interaction 2.0” and “Host–Pathogen-Interaction 3.0”, to provide an overview about various aspects of pathogenic microorganisms (viruses, bacteria, fungi and protozoa) and their interaction with their host organisms. In fact, contributions to “Host–Pathogen Interaction 3.0” and the preceding Special Issues on this subject may be divided into different topics: (i) characterization of model systems, (ii) analysis of bacteria–host interactions, (iii) analysis of viral compounds, and (iv) characterization of fungal and protozoal pathogenicity. 

Invertebrate model systems provide a cost effective and ethically unproblematic alternative to animal trials, especially in the beginning of a research project to gain first insights in the respective pathosystem or in high-throughput studies. A prerequisite is a proper characterization of the model. One of the best investigated model systems in this respect is the nematode *Caenorhabditis elegans* due to advantageous properties such as short life span, transparency, genetic tractability and ease of culture. Interestingly, Guo et al. [1] showed that plasmids of its standard bacterial diet *Escherichia coli* can interfere with nematode metabolism, an unexpected observation, which may also have an impact on studies of pathogenic bacteria. Other established model systems are protozoa such as *Acanthamoeba castellanii*, which was used to study *Salmonella enterica* sv. Typhimurium [2], the yellow mealworm *Tenebrio molitor*, which was analyzed in respect to antimicrobial peptide production in several of the submitted manuscripts [3,4,5], and larvae of the greater wax moth *Galleria mellonella* [6].

Invertebrate model systems are especially valuable for the analysis of bacterial pathogens. In fact, studies describing bacterium-host interaction were the majority of submissions to the abovementioned Special Issues. Reviews submitted summarize our current knowledge on important pathogens such as *Corynebacterium diphtheriae* [7], *Staphylococcus aureus* [8,9], *Legionella* species [10] and *Neisseria meningitidis* [11], while research articles highlight specific aspects of host–pathogen interaction. Different mycobacterial pathogens [12,13], *Corynebacterium* species [14,15], *Helicobacter pylori* [16], *Mycoplasma fermentans* [17], rickettsiae [18] and *S. enterica* [19] were studied in respect to their interaction with human cell lines. In addition to other human pathogens [20,21,22,23,24], animal- [25,26] and plant-pathogenic bacteria [27,28,29,30] were investigated.

In addition to pathogenic bacteria, fungi are important pathogens, especially in respect to agricultural plant diseases. Papers dealing with *Fusarium* infection of wheat [31] and flax [32], crop infection by *Rhizoctonia solani* [33], grapevine infection by *Lasiodiplodia theobromae* [34] and the role of fatty acid synthesis in the infection process by *Magnaporthe oryzae* [35] were submitted.

In addition to bacterial and fungal pathogens, plants are affected by viral diseases. Pathogenicity factors of Tobacco Curly Shoot Virus and Tobacco Mosaic Virus were studied in manuscripts submitted to the abovementioned Special Issues [36,37]. Of course, humans are also infected by viruses. Prominent examples are SARS-CoV-2 [38,39], influenza [40,41], Ebola virus [42] and human papillomavirus [43]. Other papers submitted to this Special Issue serial are focusing on viruses infecting animals such as pigs [44] and ducks [45].

Taken together, this serial of Special Issues provides an excellent overview about various aspects of viral, bacterial and eukaryotic pathogens and their interactions with their respective human, animal and plant hosts. The fact that several Special Issues were already successfully launched in the *International Journal of Molecular Sciences* emphasizes the scientific interest and importance of this research field at the interface of molecular biology and medicine.
